# Differences in the Gut Microbiome of Women With and Without Hypoactive Sexual Desire Disorder: Case Control Study

**DOI:** 10.2196/25342

**Published:** 2021-02-25

**Authors:** Guanjian Li, Weiran Li, Bing Song, Chao Wang, Qunshan Shen, Bo Li, Dongdong Tang, Chuan Xu, Hao Geng, Yang Gao, Guanxiong Wang, Huan Wu, Zhiguo Zhang, Xiaofeng Xu, Ping Zhou, Zhaolian Wei, Xiaojin He, Yunxia Cao

**Affiliations:** 1 Reproductive Medicine Center Department of Obstetrics and Gynecology The First Affiliated Hospital of Anhui Medical University Hefei China; 2 The First Affiliated Hospital of Anhui Medical University Hefei China; 3 National Health Commission Key Laboratory of Study on Abnormal Gametes and Reproductive Tract Hefei China; 4 Key Laboratory of Population Health Across Life Cycle Ministry of Education of the People’s Republic of China Hefei China; 5 Anhui Province Key Laboratory of Reproductive Health and Genetics Hefei China; 6 Taikang Tongji Hospital Wuhan China; 7 Biopreservation and Artificial Organs Anhui Provincial Engineering Research Center Hefei China

**Keywords:** gut microbiome, metabolome, sexual desire, online recruitment, biomarkers

## Abstract

**Background:**

The gut microbiome is receiving considerable attention as a potentially modifiable risk factor and therapeutic target for numerous mental and neurological diseases.

**Objective:**

This study aimed to explore and assess the difference in the composition of gut microbes and fecal metabolites between women with hypoactive sexual desire disorder (HSDD) and healthy controls.

**Methods:**

We employed an online recruitment method to enroll “hard-to-reach” HSDD populations. After a stringent diagnostic and exclusion process based on DSM-IV criteria, fecal samples collected from 24 women with HSDD and 22 age-matched, healthy controls underwent microbiome analysis using 16S ribosomal RNA gene sequencing and metabolome analysis using untargeted liquid chromatography–mass spectrometry.

**Results:**

We found a decreased abundance of *Ruminococcaceae* and increased abundance of *Bifidobacterium* and *Lactobacillus* among women with HSDD. Fecal samples from women with HSDD showed significantly altered metabolic signatures compared with healthy controls. The abundance of *Bifidobacterium*, *Lactobacillus*, and several fecal metabolites correlated negatively with the sexual desire score, while the number of *Ruminococcaceae* correlated positively with the sexual desire score in all subjects.

**Conclusions:**

Our analysis of fecal samples from women with HSDD and healthy controls identified significantly different gut microbes and metabolic signatures. These preliminary findings could be useful for developing strategies to adjust the level of human sexual desire by modifying gut microbiota.

**Trial Registration:**

Chinese Clinical Trial Registry ChiCTR1800020321; http://www.chictr.org.cn/showproj.aspx?proj=34267

## Introduction

Hypoactive sexual desire disorder (HSDD) is defined as a deficiency or absence of desire for sexual activity that causes marked distress or interpersonal difficulty and is not accounted for by another psychiatric condition, use of medications, or relationship problems [1,2]. Two large epidemiological surveys have shown that this combination of symptoms, “low desire and associated distress,” is present in up to 10% of American women, with similar prevalence rates seen throughout the world [3,4]. HSDD is the most frequently reported female sexual health problem, and women with HSDD may experience reduced quality of life, impaired physical image, and decreased feelings of self-confidence and self-worth, in addition to feeling less connected with their partners and families [5].

In humans, sexual desire is regulated by key areas of the brain through the action of various neurotransmitters [6,7]. Norepinephrine, dopamine, melanocortin, oxytocin, and vasopressin mediate sexual excitation, while serotonin, opioids, prolactin, and the endogenous cannabinoid system mediate sexual inhibition. Generalized HSDD may be related to a neuropsychological state of increased inhibition or decreased excitation, or a mixture of the two [8,9]. Neurotransmitters in the central nervous system are thus therapeutic targets for improving human sexual desire. At present, the main treatment strategies are to reduce the action of 5-HT, enhance the action of dopamine, or both [10].

Human beings exist in a mutualistic relationship with their gut microbiota, a highly diverse and complex microbial ecosystem closely associated with various phenotypes and diseases [11,12]. Perturbations in gut microbe richness and diversity affect the levels of 5-HT, norepinephrine, and γ-aminobutyric acid (GABA)ergic and dopaminergic neurotransmission in the brain [13-15]. These pathways and molecules are believed to be closely related to human sexual desire.

Although this evidence suggests that there may be a connection between the composition of gut microbiota and sexual desire, our knowledge of the role of gut microbiota in sexual desire disorders is limited. We hypothesized that the fecal gut microbiota and metabolites of patients with sexual desire disorders differ from those of people without such disorders and that active metabolites and neurochemicals may be mediators between gut microbes and the sexual desire system.

Recruiting participants for microbial research is increasingly difficult as the number of projects competing for participants’ attention increases and response rates decline. In addition, women with desire disorder constitute a hard-to-reach population owing to fear and lack of trust in the study procedures or in research staff, especially in a more conservative society like China when compared with Western countries [16]. Widespread access to online tools now offers various advantages in data collection that can reduce administrative procedures and improve information privacy [17,18]. Previous studies have demonstrated that performance and effectiveness obtained using online recruitment methods in studies of sexual issues or gut microbes concur with those obtained using offline recruitment methods, such as telephone surveys, mail-in questionnaires, posters, and flyers [12,19-21]. In addition, online surveys may even provide greater reach and be less expensive, while allowing for convenient data collection and analysis [22-24]. These findings suggest that the growth of the internet and social media sites may provide new opportunities for research on the correlation between sensitive health concerns (such as mental health problems or sexual health disorders) and gut microbes.

We conducted this study to investigate the composition of the gut microbiome and metabolite abundance in women with HSDD and to increase our understanding of the possible association between human sexual desire and gut microbiota and fecal metabolites.

## Methods

### Participants

From March 2019 to November 2019, we recruited subjects by posting advertisements on instant messenger applications and the information platform. In all advertisements, potential participants were automatically redirected to a study website that included a link to the survey and participant information including study details and information statement.

Potential recruits included women who had previously been diagnosed with suspected HSDD but not yet treated or who complained of decreased sexual desire, causing them to seek medical counseling, as well as volunteers who claimed to have normal sexual desire. No incentives were offered to responders. When requested, they were provided a brief report on their own gut microbial composition. All responders provided electronic written informed consent. All experimental protocols used in this study were approved by the Ethics Committee of Anhui Medical University and the relevant hospitals.

### Clinical Information Collection

We collected information online from 157 premenopausal women and scheduled face-to-face diagnostic interviews and biological sample collection to identify women with generalized acquired HSDD. Incomplete questionnaires and responses that rejected further face-to-face interviews were not accepted by the system. A combination of a web-based questionnaire and structured interviews was used to collect information on demographic characteristics such as ethnicity, marital status, income, educational level, reproductive history, sexual partner relationships, sexual frequency, sexual dysfunction, and the presence and treatment of related diseases.

### 16S Ribosomal RNA Gene Sequencing and Metabolomics Tests

Subsequently, we performed high-throughput 16S ribosomal RNA gene sequencing and untargeted liquid chromatography–mass spectrometry metabolomic analysis of stool samples from the 26-member HSDD cohort and 26 healthy controls (for all survey measures and analysis methods, see the Methods section in Multimedia Appendix 1).

## Results

### Clinical Characteristics of the Subjects

**Figure 1:**
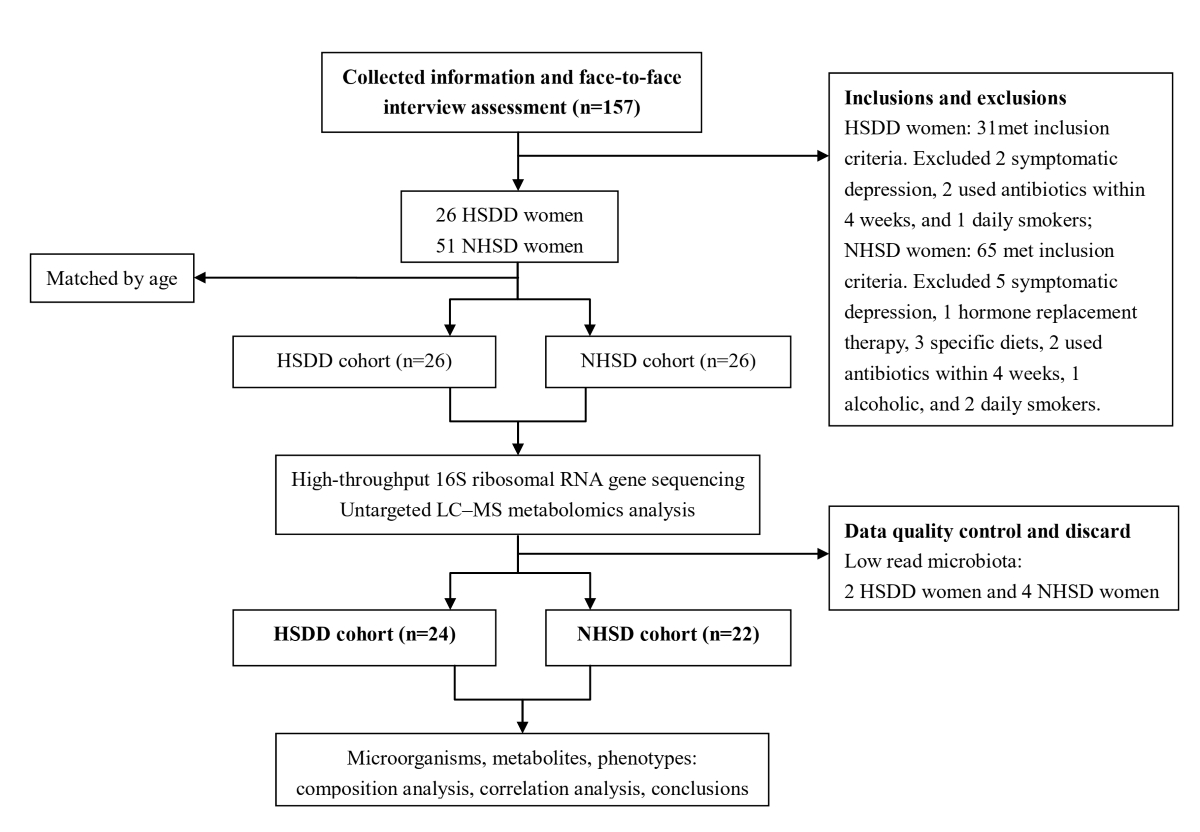
Inclusion and exclusion processes. HSDD: hypoactive sexual desire disorder; LC-MS: liquid chromatography–mass spectrometry; NHSD: no history of sexual dysfunction.

A flowchart showing the inclusion and exclusion processes is presented in Figure 1. In the end, 24 women with HSDD and 22 women with no history of sexual dysfunction (NHSD) were included in the final analyses. The clinicopathological variables of the HSDD and NHSD cohorts are shown in Table 1. All participants were Han Chinese from the Hefei area and had comparable geographical conditions and eating habits. The clinicopathological variables of the HSDD and NHSD cohorts were comparable, except for a slight increase in platelet count in the HSDD cohort (the meaning is unclear). Significant differences were observed in Female Sexual Function Index desire domain scores between the HSDD and NHSD groups.

**Table 1:** Demographic and other sample data at baseline.

Characteristic	HSDD^a^ (n=24)	NHSD^b^ (n=22)	*P* value
Age (years), mean (SD)	33.8 (4.5)	33.9 (4.0)	.91
BMI (kg/m^2^), mean (SD)	27.1 (3.6)	26.3 (3.3)	.27
College or beyond, n	17	16	.89
FSFI-D^c^ score, mean (SD)	3.2 (0.9)	8.6 (1.0)	.01
CES-D^d^ score, mean (SD)	10.3 (4.9)	10.9 (4.6)	.55
RBC^e^ (×10^12^/L), mean (SD)	4.3 (0.7)	4.1 (0.4)	.06
WBC^f^ (×10^9^/L), mean (SD)	6.9 (1.7)	6.5 (1.7)	.28
PLT^g^ (×10^12^/L), mean (SD)	169.4 (44.8)	154.0 (35.9)	.04
FSH^h,i^ (mIU/mL), mean (SD)	6.3 (3.0)	6.3 (2.9)	.93
LH^i,j^ (mIU/mL), mean (SD)	5.3 (3.4)	5.9 (3.7)	.67
Estradiol^i^ (pg/mL), mean (SD)	48.1(9.3)	50.6 (6.5)	.09
Total T^i,k^ (ng/mL), mean (SD)	0.5 (0.3)	0.5 (0.2)	.86

^a^HSDD: hypoactive sexual desire disorder.

^b^NHSD: no history of sexual dysfunction.

^c^FSFI-D: Female Sexual Function Index desire domain.

^d^CES-D: Center for Epidemiologic Studies Depression Scale.

^e^RBC: red blood cell.

^f^WBC: white blood cell.

^g^PLT: platelet.

^h^FSH: follicle-stimulating hormone.

^i^Due to missing data, the group sizes differ for hormonal data: HSDD group, n=20; NHSD group, n=14.

^j^LH: luteinizing hormone.

^k^T: testosterone.

### HSDD and Gut Microorganisms

**Figure 2:**
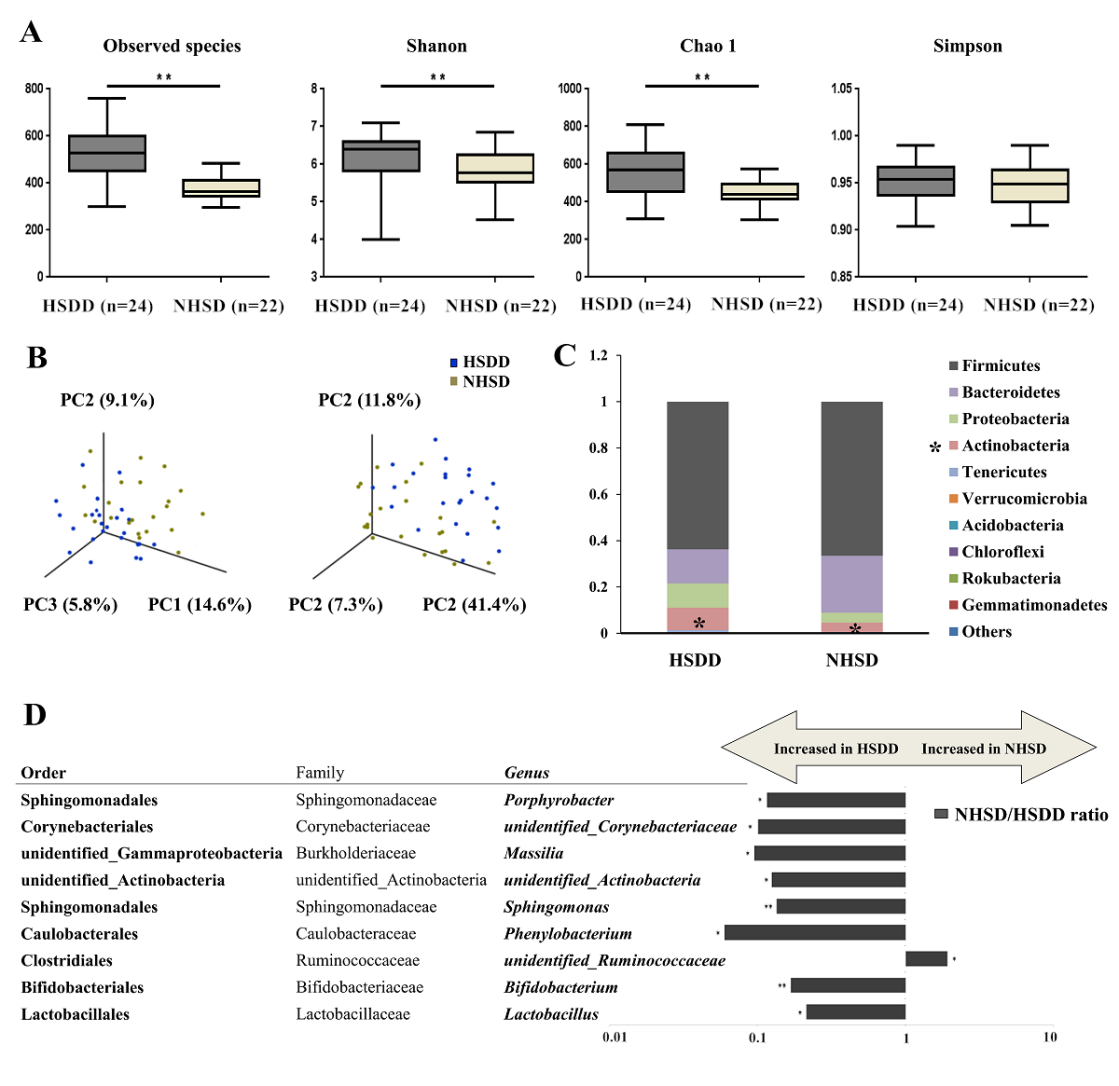
Gut microbial characteristics of women with hypoactive sexual desire disorder (HSDD) or with no history of sexual dysfunction (NHSD), based on (A) α-diversity analysis of 3 indexes (observed species, shannon, and Chao 1); (B) β-diversity analysis, providing unweighted and weighted principal coordinate (PC) plots; (C) component proportion of bacterial phylum in each group; (D) the ratio between HSDD and NHSD on the logarithmic scale. All QFDR<0.05.

In our investigation, 1753 operational taxonomic units were identified from sequenced specimens with 97% sequence similarity. Different operational taxonomic unit–based diversity indexes were used to assess the bacterial composition within each sample (the α-diversity). Compared to the NHSD group, the HSDD cohort was characterized by a higher number of observed species and higher Shannon and Chao 1 indexes (Figure 2A, except for Simpson), suggesting greater species diversity in gut microbiota in the HSDD cohort. β-diversity analysis was performed to determine whether HSDD was associated with altered gut microbial composition. As shown in Figure 2B, both unweighted and weighted principal coordinate analysis plots revealed that the gut microbial composition of the HSDD group was significantly different from that of the NHSD group.

At the phylum level, the most abundant taxa were *Firmicutes* and *Bacteroidetes*, followed by *Proteobacteria* and *Actinobacteria* in the exploration cohort (Figure 2C). *Actinobacteria* were significantly enriched in the HSDD group compared to the NHSD group (9.1% versus 3.3%, *P*<.01). At the genus level, 9 taxa were different between the HSDD and NHSD groups (Figure 2D). *Bifidobacterium*, *Lactobacillus*, and *Sphingomonas* were enriched in women with HSDD, while an unidentified genus of *Ruminococcaceae* was enriched in NHSD women (*P*<.05 and QFDR<0.20). *Lactobacillus* and *Ruminococcaceae* belong to the same phylum, *Firmicutes*, which is the most abundant phylum, while *Bifidobacterium* belong to the phylum *Actinobacteria*.

### HSDD and the Fecal Metabolome

**Figure 3:**
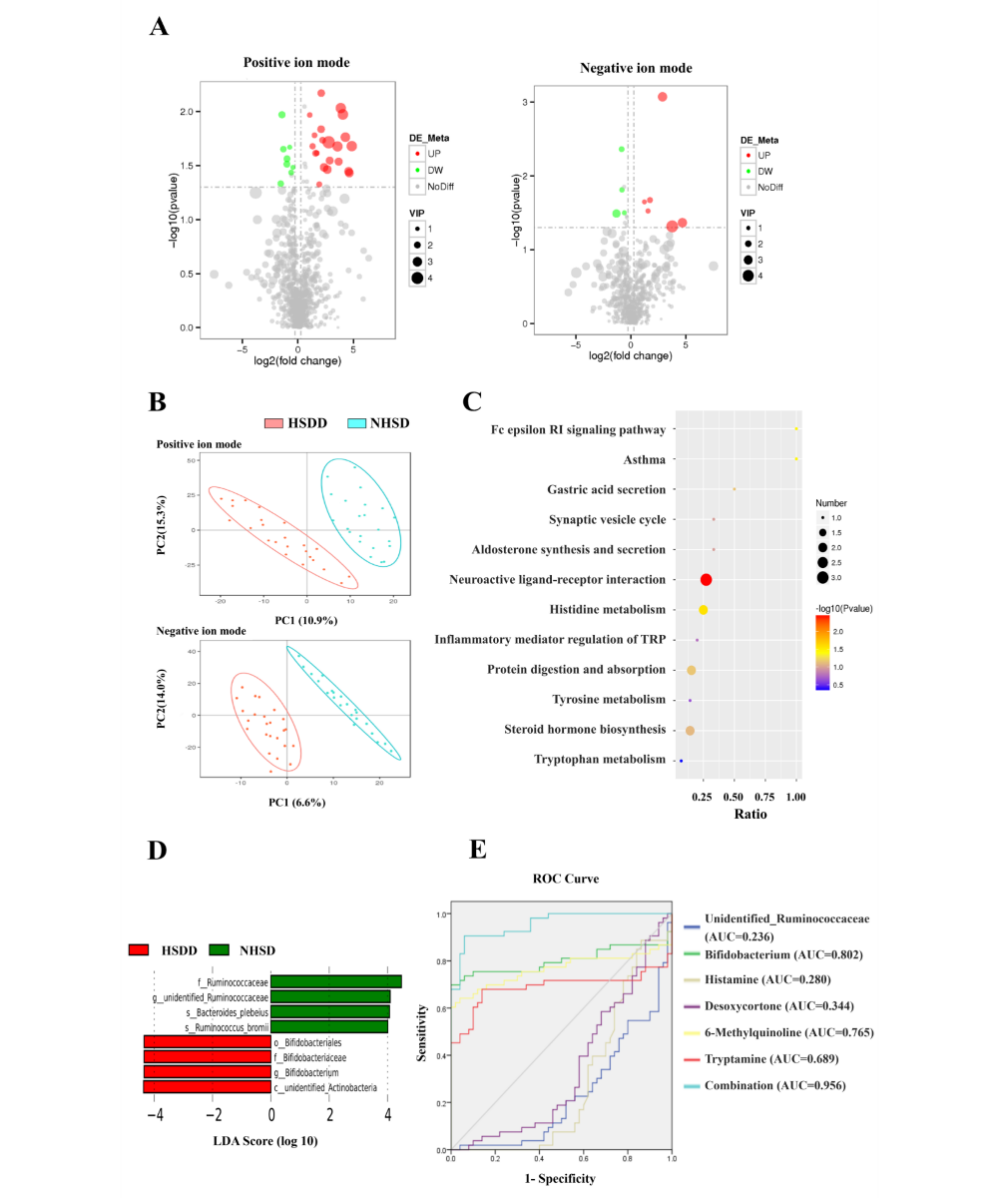
Fecal metabolites characteristics of women with hypoactive sexual desire disorder (HSDD) and women with no history of sexual dysfunction (NHSD), displayed as (A) volcano plots showing the differentially accumulated (log2[fold change] and significantly changed (-log10[pvalue] fecal metabolites; (B) principal component (PC) analysis showing grouped discrimination; (C) functions and pathways of these metabolites using the KEGG database, where the differential metabolites are annotated on 12 different KEGG metabolic pathways; (D) Lefse and linear discriminant analyses showing differences in taxonomic composition; (E) receiver operating characteristic (ROC) curve. AUC: area under the curve; TRP: tryptophan.

In the fecal metabolome analysis, 1168 metabolites were quantified. Differential metabolite volcanic maps show that the fecal metabolic phenotype of women with HSDD was significantly different than that of women with NHSD (Figure 3A). Principal component analysis clearly showed differences between the HSDD and NHSD groups, indicating that women with HSDD have specific metabolic abundances and characteristics (Figure 3B). In total, 28 metabolites that made major contributions to the distinction (VIP score >1.5) were selected using partial least squares discriminant analysis. These differential metabolites were annotated to 12 different KEGG metabolic pathways [25], including neuroactive ligand-receptor interactions, the Fc epsilon RI signaling pathway, steroid hormone biosynthesis, histidine metabolism, and tryptophan metabolism (Figure 3C).

### A Panel of Fecal Microorganisms and Metabolic Markers Identifies HSDD

To further identify the specific bacterial components associated with HSDD, we used a standard microbiota analysis tool, LefSe; 8 taxa were identified as key biomarkers (*P*<.05, linear discriminant analysis scores >4). Several taxa, including the genus *Bifidobacterium* and class unidentified *Actinobacteria*, were significantly overrepresented in the HSDD women, whereas the genus unidentified *Ruminococcaceae* was overrepresented in the NHSD women (Figure 3D).

To identify and quantify the potential of microbial and metabolic biomarkers for HSDD, we performed a receiver operating characteristic curve analysis using the relative abundance of the *Bifidobacterium* genus and the unidentified_*Ruminococcaceae* genus, the most abundant genus identified, to distinguish HSDD from NHSD. Then, a binary logistic regression analysis for the 2 genera and 28 significantly correlated metabolites was performed. Significant deviations between women with HSDD and women with NHSD could be identified using *Bifidobacterium* and unidentified_*Ruminococcaceae* at the genus level, as well as 4 significantly correlated metabolites: histamine (*P*=.01), desoxycortone (*P*=.03), 6-Methylquinoline (*P*=.01), and tryptamine (*P*=.03). This diagnostic marker panel (combination of 2 microbial markers and 4 fecal metabolites) discriminated between the HSDD and NHSD groups with 95.6% accuracy (area under the curve=0.956; Figure 3E).

### Relationships Between Gut Microbiota, Metabolites, and Clinical Characteristics

A Spearman’s rank correlation test was performed to explore the correlations between gut microbes, fecal metabolites, and clinical characteristics. The altered bacterial genera were generally associated with differential metabolites (Multimedia Appendix 2), and almost all the differential bacterial genera showed significant correlations with a range of differential fecal metabolites (*P*<.05). Spearman correlation analysis between every 2 variables suggested that some bacterial genera and fecal metabolomes were slightly associated with clinical characteristics such as age, BMI, Center for Epidemiologic Studies Depression Scale score, and some blood indicators (Multimedia Appendix 2). We found that some genera and metabolites were significantly correlated with the Female Sexual Function Index desire domain score (|r|>0.35, *P*<.05), providing strong evidence for the existence of a correlation between sexual desire and gut microorganisms.

## Discussion

### Principal Findings

These results suggest significant differences in the composition of fecal microbiota between controls and women with HSDD. We observed a significantly higher quantity of *Actinobacteria* in women with HSDD compared with healthy controls. The altered abundance of several genera contributed to the unique gut microbial characteristics found in HSDD. The most prominent of these was the marked enrichment of the *Bifidobacterium* genus. Correlation analysis showed that the greater number of *Bifidobacterium* and *Lactobacillus* could be associated with a decrease in sexual desire. Higher microbial diversity has been shown in previous studies to have benefits for the human body, and elevated levels of *Bifidobacterium* and *Lactobacillus* have been related to reduced anger, dysphoric mood, aggressive thoughts, and self-reported feelings of sadness [26,27]. A possible explanation for these observations is that these emotions and human sexual desires are physiologically interrelated, involving a variety of neurotransmitters and other changes in brain biochemistry. Anger, fear, or stress can be a prelude to sex, specifically because these emotional states generate enthusiasm or excitement that leads to an “excitement transfer” into sexual desire and arousal [28].

There is considerable evidence that gut microorganisms modulate the metabolism of dopamine, 5-HT, and noradrenaline in the brain [15,29,30]. These molecules are generally considered to be the major neurotransmitters regulating sexual desire [7]. Previous research suggests that plasma concentrations of kynurenic acid and tryptophan (serotonergic precursor) in Sprague-Dawley rats treated with *Bifidobacterium* for 14 days increased significantly compared with controls. *Bifidobacterium* treatment also resulted in a decrease in 5-hydroxyindoleacetic acid concentration in the frontal cortex and dihydroxyphenylacetic acid concentration in the amygdala [31]. In another study, rats were orally administered specific *Bifidobacterium* every day, and *Bifidobacterium* reportedly produced GABA through glutamate decarboxylase [32]. *Ruminococcaceae*, also commonly referred to as Clostridia clusters XIVa and IV, are abundant microorganisms in the normal gut. They decompose indigestible carbohydrates and produce short-chain fatty acids (SCFAs). Numerous studies have shown that SCFAs, including butyrate, can cross the blood-brain barrier and enter the central nervous system and may play a role in neuropsychiatric conditions and general psychological functions [33]. SCFAs might directly influence the brain by influencing the blood-brain barrier, stimulating the vagus nerve, regulating the secretion of neurotrophic factors, modulating 5-HT/dopamine/noradrenaline/GABA biosynthesis, and promoting the transcription of inhibitory pathway transcripts [34]. These findings lead to the hypothesis that neurotransmitter molecules and pathways may play crucial roles in the regulation of sexual desire by gut microorganisms.

The analysis of fecal metabolic components has received considerable attention since these biomolecules reflect genetic and environmental effects and also act to connect the health of the host and its symbiotic microorganisms [35]. In fecal samples from women with HSDD, metabolites had significantly different distributions from the control group. Enriched functional modules included those involved in neuroactive ligand-receptor interactions, histidine metabolism, steroid hormone biosynthesis, and tryptophan metabolism.

The systematic exclusion of alternative diagnoses by structured interviews and the use of rigorous differential diagnosis criteria based on the DSM-IV resulted in a representative cohort of individuals with generalized acquired HSDD, avoiding various genital disorders existing in men or elderly women, rather than the sexual desire problem of “central effect.” In addition, although we reported Center for Epidemiologic Studies Depression Scale 20 scores, estradiol, testosterone, and sex hormone-binding globulin, in fact, the cause of HSDD is multifactorial and variable; changes in hormones, stress, depression, present sexual experiences, past history of abuse, total well-being, sexual condition of the sexual partner, and relationship factors may also be crucial causes of sexual desire disorder [36,37]. Therefore, whether our findings might be specific to HSDD or more general changes seen in multiple disorders need to be further explored in future studies with large samples.

Given the increased popularity of online media and information platforms, many researchers are using web-based tools to recruit participants for medical, psychological, and sociological research studies (especially for stigmatized and hard-to-reach groups). Previous studies have shown that online methods can successfully recruit and manage people who are humiliated or discriminated against because of race, sexual orientation, or mental health status [17,18]. Our results confirmed recent literature regarding online recruitment of hard-to-reach people and expanded the scope of recruitment and management to the study population of sexual dysfunction and gut microbe research.

### Limitations

There are several potential limitations of our research. First, our online recruitment methods relied on convenience samples, and determining the source population limits the external validity of findings. When it comes to sensitive issues, determining adequate compensation and ensuring confidentiality continue to be important concerns. Second, gut microorganisms and metabolites are affected by a variety of factors, including geography, daily diet, and genetic variation [38]. Additionally, race and ethnic culture appear to have a significant association with the occurrence of sexual problems [39]. Therefore, since all the participants were from a single ethnicity, identifying specific correlations between gut microorganisms and sexual desire in a global population may not be straightforward. Another issue is that there is no standardized method for adjusting the composition of the microbiome using diet. In this study, this issue was handled by excluding all individuals who reported variations in their diet, such as binge eating or purging, following a vegetarian diet, or having gluten or lactose intolerance. However, it cannot be ruled out that subtle, unspecified dietary factors could have influenced our results. Finally, our findings only demonstrated an association between the gut microbiome and HSDD. Whether alternations in the gut microbiome are responsible for HSDD should be further validated using animal models in the laboratory.

### Conclusions

This study addressed a number of gaps. We identified numerous microbial genera, including *Bifidobacterium* and *Lactobacillus*, that were enriched in women with HSDD. In addition, our metabolomic analysis of fecal samples from women with HSDD and healthy controls identified significantly different metabolic signatures. Our results support the hypothesis that disturbances in gut microorganisms are closely related to the level of human sexual desire. Pathogenicity of the microbiome in women with HSDD may be caused by comprehensive changes in holistic interaction, rather than by specific pathogens or metabolites.

A better understanding of the microbiota’s role in the gut-brain-libido axis may lead to the identification of novel drug targets and management measures to improve sexual health. Much work remains to be performed in determining the role and mechanisms of the gut microbiota in human sexual desire.

## Appendix

Multimedia Appendix 1 Supplementary methods.

Multimedia Appendix 2 Heat map. (a) Heat map of the Spearman’s rank correlation coefficient of differential bacterial genus, differential metabolites and HSDD clinical characteristics. (b) Heatmaps showing altered bacterial genus were generally associated with differential fecal metabolites. The statistical significance was denoted on the squares (#p < 0.05; ##< 0.01).

## Abbreviations

GABA: γ-aminobutyric acid
HSDD: hypoactive sexual desire disorder
NHSD: no history of sexual dysfunction
SCFA: short-chain fatty acids
