# Supplementary material for: Differences in the Gut Microbiome of Women With and Without Hypoactive Sexual Desire Disorder: Case Control Study
Source: J Med Internet Res. 2021 Feb 25;23(2):e25342. doi: 10.2196/25342 (PMC7952237; doi:10.2196/25342)
Supplement: Multimedia Appendix 1 [file jmir_v23i2e25342_app1.docx]

# Supplementary methods

A combination of baseline computer-administered self-interview questionnaire and face-to-face structured interviews was used to collect information on demographic characteristics such as ethnicity, marital status, income, and educational level. We also collected data on reproductive history, sex partner relationships, sexual frequency, sexual dysfunction, and the presence and treatment of related diseases. We selected participants who had been in steady heterosexual relationships for three months or longer, so that the women would have ample opportunities for sexual intercourse and partner factors in the sexual relationship were more assessable.

**Clinical assessment**

In the morning after day three of the menstrual cycle 20 ml of venous blood was drawn from the fasting subjects. Of this sample, 10 ml was analyzed using routine blood and biochemical indexes within one hour. Another 10 ml was used to measure hormone levels using chemiluminescence immunoassays. Operating instructions were strictly observed during all processes.

A standardized, validated diagnostic interview assessment of HSDD status is the most important component in the diagnosis of HSDD. All interviews were conducted by experienced, trained doctors or sexual medicine experts according to strict diagnostic assessment guidelines. Three validated instruments were used in the assessment: the Decreased Sexual Desire Screener (DSDS) to diagnose generalized acquired HSDD; the Female Sexual Function Index (FSFI) to assess sexual function in women; and the Center for Epidemiological Studies Depression Scale (CES-D) to screen for depressive disorders in patients.

The CES-D scale, which consists of 20 items, is one of the commonly used measures for depressive disorders. In order to screen for current depression, participants with CES-D 20 scores of 16 or higher  were categorized as having symptomatic depression, and were excluded from the study[1]. The FSFI is a widely used 19-item questionnaire designed to measure the main dimensions of female sexual function. It has been validated, and is believed to reliably discriminate between women suffering from sexual dysfunction and healthy women[2]. The Chinese version of the FSFI, the CVFSFI, is a dependable, well-structured screening questionnaire which was validated by Sun et al. in 2011[3]. The original score on each FSFI domain ranges from 0 to 5 or 1 to 5. The desire domain questionnaire (FSFI-D) evaluates the frequency and degree of female sexual desire. DSDS is a short, validated tool for obtaining sensitive and reliable diagnosis of generalized, acquired HSDD in women. In a recent series of validation trials, the DSDS was found to have high sensitivity and specificity in multiple female populations[4, 5]. It consists of five “yes/no” questions, the first four of which assess the symptoms of HSDD, including lack of desire and associated distress. In order to be eligible for a diagnosis of generalized, acquired HSDD, participants must answer “yes” to the first four questions. For participants who answered “yes” to the first four questions, the fifth multi-item question is used to assess relevant medical comorbidities and potential adverse effects of drugs. Further examination and differential diagnosis were carried out to determine whether a diagnosis of generalized acquired HSDD is appropriate.

The inclusion criteria for the HSDD group were: 1) age 20-45 premenopausal women, with a total FSFI-D score of 5 or less, meeting the DSDS criteria for generalized acquired HSDD; 2) having been in a stable heterosexual relationship for at least three months in the past two years; and 3) willing to provide biological samples. The criteria for exclusion were: 1) any serious or chronic medical problem (cardiovascular, hepatic, pulmonary, gastrointestinal, immunological, or endocrine); 2) any neurological or psychiatric disorder, including symptomatic depression as recognized by CES-D; 3) any other primary sexual dysfunction; 4) use of medications that might affect sexual desire and function; 5) the use of antibiotics, probiotics, or prebiotics within four weeks; 6) specific diets (vegetarian, gluten or lactose intolerance, binge eating); 7) pregnant or lactating; 8) a history of significant alcoholism, drug abuse, or daily smoking; 9) incomplete or unqualified of questionnaire information, stool or blood samples; or 10) lack of research authorization. The group with no history of sexual dysfunction (NHSD) comprised 20-45 year old premenopausal women with normal sexual function who were age-matched with the HSDD group. The exclusion criteria for the NHSD group were the same as those for the HSDD group.

**Gut microbiome composition and metabolite**

Fecal samples of all participants were collected after two or three days of the menstrual cycle in our medical center and stored at -80℃(packed into two frozen pipes) before DNA was extracted or metabolomic profiling took place. The V3-V4 regions of the 16S rRNA genes were amplified used specific primer with the barcode. All PCR reactions were carried out with 15 µL of Phusion High-Fidelity PCR Master Mix (New England Biolabs). After purification (Qiagen Gel Extraction Kit, Germany), Sequencing libraries were generated usingTruSeq DNA PCR-Free Sample Preparation Kit (Illumina, USA) following manufacturer's recommendations and index codes were added. The library quality was assessed on the Qubit 2.0 Fluorometer (Thermo Scientific, China). At last, the library was sequenced on an Illumina NovaSeq platform and 250 bp paired-end reads were generated.

The pairedend reads were generated and assigned to sample based on their unique barcodes and then were merged with FLASH (V1.2.7) software. High quality filtering of the raw tags were conducted to acquire clean tags according to the QIIME(V1.9.1) The tags were compared with the reference database using the UCHIME Algorithm to detect and remove chimera sequences, and obtain effective tags. The chimeric sequences analysis were filtered using Uparse software (V7.0.1001). Sequences with ≥97% similarity were assigned to the same OTUs. The Silva Databasebased on the Mothur algorithm was used to annotate the taxonomic information. OTUs whose sequence number less than 0.005% of total sequences number were discarded as recommended. Samples with reads less than 8000 were also discarded.

The extracted tissues were grounded with liquid nitrogen, and the homogenate was resuspended with prechilled 80% methanol and 0.1% formic acid. The samples were incubated on ice for 5 minutes, then centrifuged. Some supernatants were diluted with LC-MS grade water to final concentration containing 53% methanol. Centrifugation again, the supernatant was injected into LC-MS/MS system for analysis. LC-MS analyses were performed using an Orbitrap Q Exactive series mass spectrometer (Thermo Fisher) and a Vanquish UHPLC system (Thermo Fisher). Samples were injected into Hyperil Gold column with a linear gradient of 16min. The positive polarity mode eluents were eluent A (0.1% fatty acid in water) and eluent B (Methanol). The negative mode eluents were eluent A (pH 9.0, 5 mM ammonium acetate) and eluent B (Methanol). Q Exactive series mass spectrometer adopts positive/negative polarity mode, spray voltage 3.2 kV, capillary temperature 320°C, sheath gas flow rate of 35 arb, aux gas flow rate of 10 arb. For each metabolite, Compound Discoverer 3.1 (CD3.1, Thermo Fisher) was used to process the raw data files generated by UHPLC-MS/MS for peak alignment, peak picking, and quantitation. Based on additive ions, molecular ion peaks and fragment ions, normalized data were used to predict molecular formula. MzCloud, mzVault and MassList database were used to match the peaks to obtain accurate qualitative and relative quantitative results.

**Statistical analyses**

Software R (R version R 3.4.3), SPSS (SPSS, Chicago, IL, US) and Graph Pad Prism 6 (Graph Pad Software, CA, USA) were used for statistical analysis. A two-tail p value of less than 0.05 was considered to be statistically significant. Student's t test and Mann–Whitney U test was applied for continuous variables, and categorical variables was performed using the χ2 test or Fisher’s exact test. Alpha diversity was compared using MannWhitney or KruskalWallis tests to identify the complexity of species diversity for each sample; beta diversity was preceded by principal coordinate analyses (PCoA). PCA (principal components analysis) and OPLS-DA were performed. FDR (False discovery rate) control was used to correct multiple tests. LDA (linear discriminant analysis) combined with LEfSe (effect size) was used to evaluate the taxa with the differences. Spearman’s rank correlation test was performed to calculate the correlation between gut microbial, metabolites and clinical characteristics.

**reference**

1. Radloff LS. The use of the Center for Epidemiologic Studies Depression Scale in adolescents and young adults. J Youth Adolesc. 1991 Apr;20(2):149-66. PMID: 24265004. doi: 10.1007/BF01537606.

2. Rosen R, Brown C, Heiman J, Leiblum S, Meston C, Shabsigh R, et al. The Female Sexual Function Index (FSFI): a multidimensional self-report instrument for the assessment of female sexual function. J Sex Marital Ther. 2000 Apr-Jun;26(2):191-208. PMID: 10782451. doi: 10.1080/009262300278597.

3. Sun X, Li C, Jin L, Fan Y, Wang D. Development and validation of Chinese version of female sexual function index in a Chinese population-a pilot study. J Sex Med. 2011 Apr;8(4):1101-11. PMID: 21235720. doi: 10.1111/j.1743-6109.2010.02171.x.

4. Clayton AH, Goldfischer ER, Goldstein I, Derogatis L, Lewis-D'Agostino DJ, Pyke R. Validation of the decreased sexual desire screener (DSDS): a brief diagnostic instrument for generalized acquired female hypoactive sexual desire disorder (HSDD). J Sex Med. 2009 Mar;6(3):730-8. PMID: 19170868. doi: 10.1111/j.1743-6109.2008.01153.x.

5. Clayton AH, Goldfischer E, Goldstein I, DeRogatis L, Nappi R, Lewis-D'Agostino DJ, et al. Validity of the decreased sexual desire screener for diagnosing hypoactive sexual desire disorder. J Sex Marital Ther. 2013;39(2):132-43. PMID: 23252638. doi: 10.1080/0092623X.2011.606496.
