# Supplementary figures and images for: Differences in the Gut Microbiome of Women With and Without Hypoactive Sexual Desire Disorder: Case Control Study
Source: J Med Internet Res. 2021 Feb 25;23(2):e25342. doi: 10.2196/25342 (PMC7952237; doi:10.2196/25342)

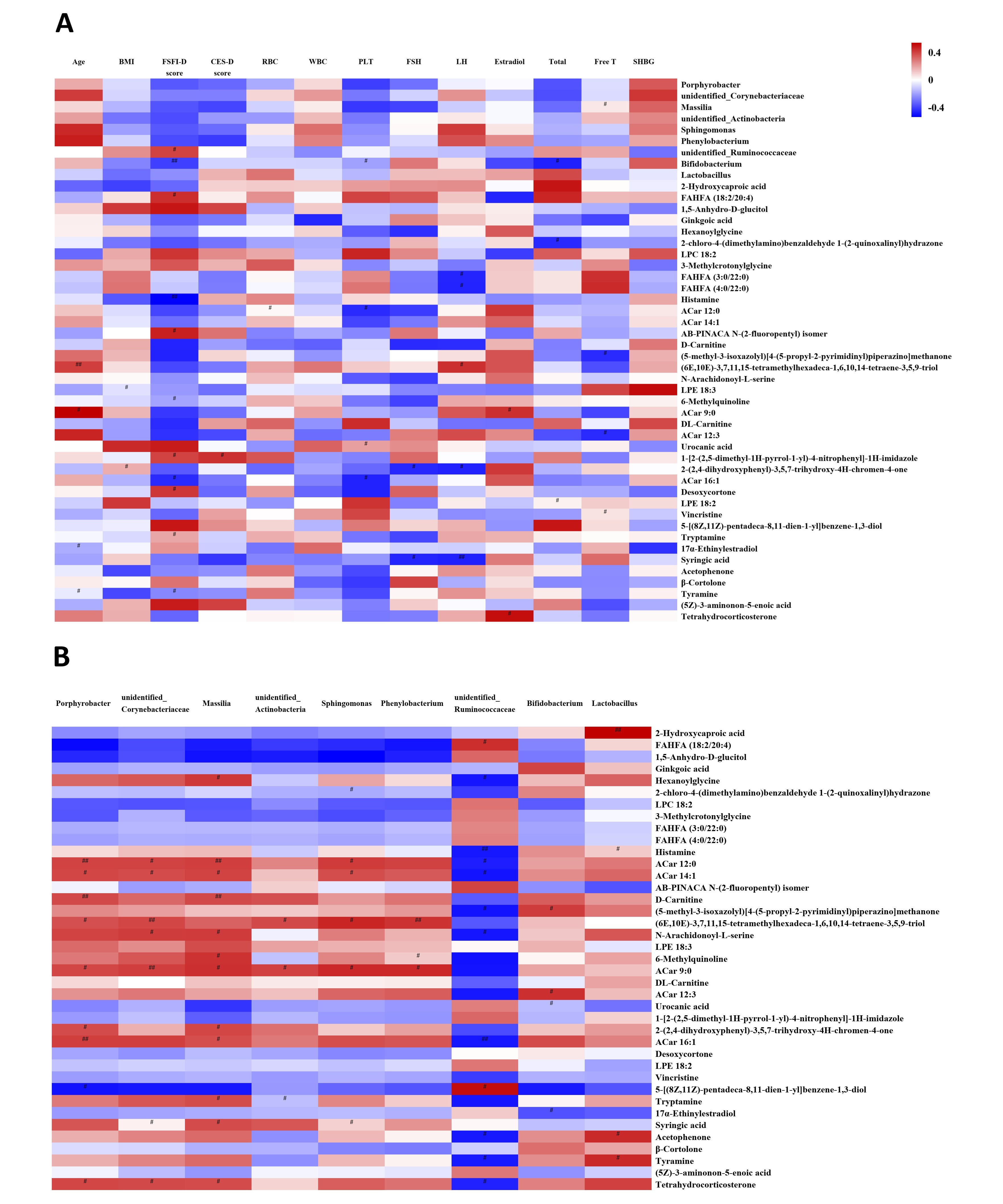

Supplement: Multimedia Appendix 2 [file jmir_v23i2e25342_app2.png]
